# Supplementary figures and images for: Integrated multi-omics analysis reveals the functional signature of microbes and metabolomics in pre-diabetes individuals
Source: Microbiol Spectr. 2025 Jun 9;13(7):e01459-24. doi: 10.1128/spectrum.01459-24 (PMC12211063; doi:10.1128/spectrum.01459-24)

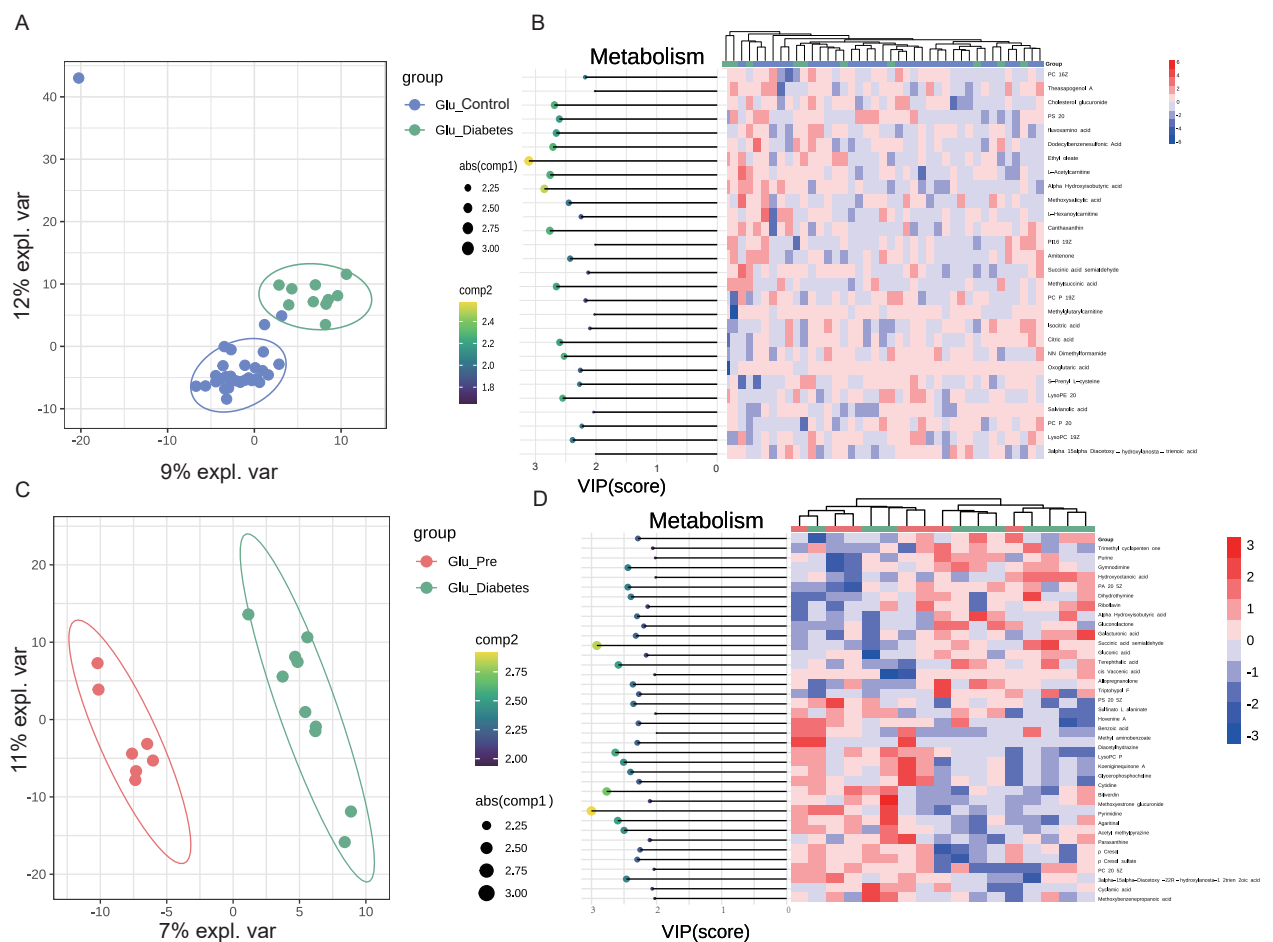

Supplement: Fig. S1 — Metabolic profile alterations among the Diabetes group, Healthy control group, and Pre-diabetes group. [file spectrum.01459-24-s0001.pdf]

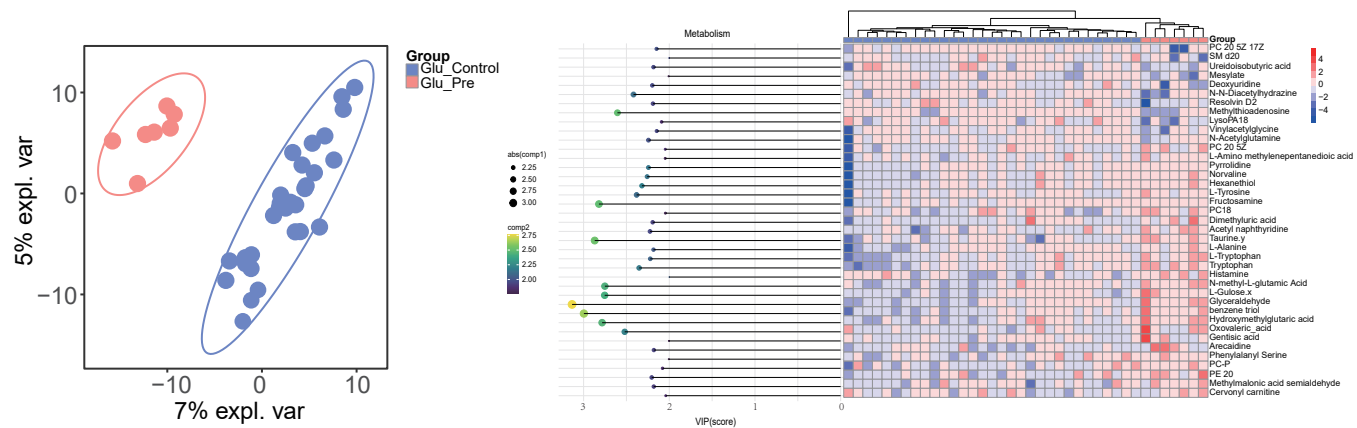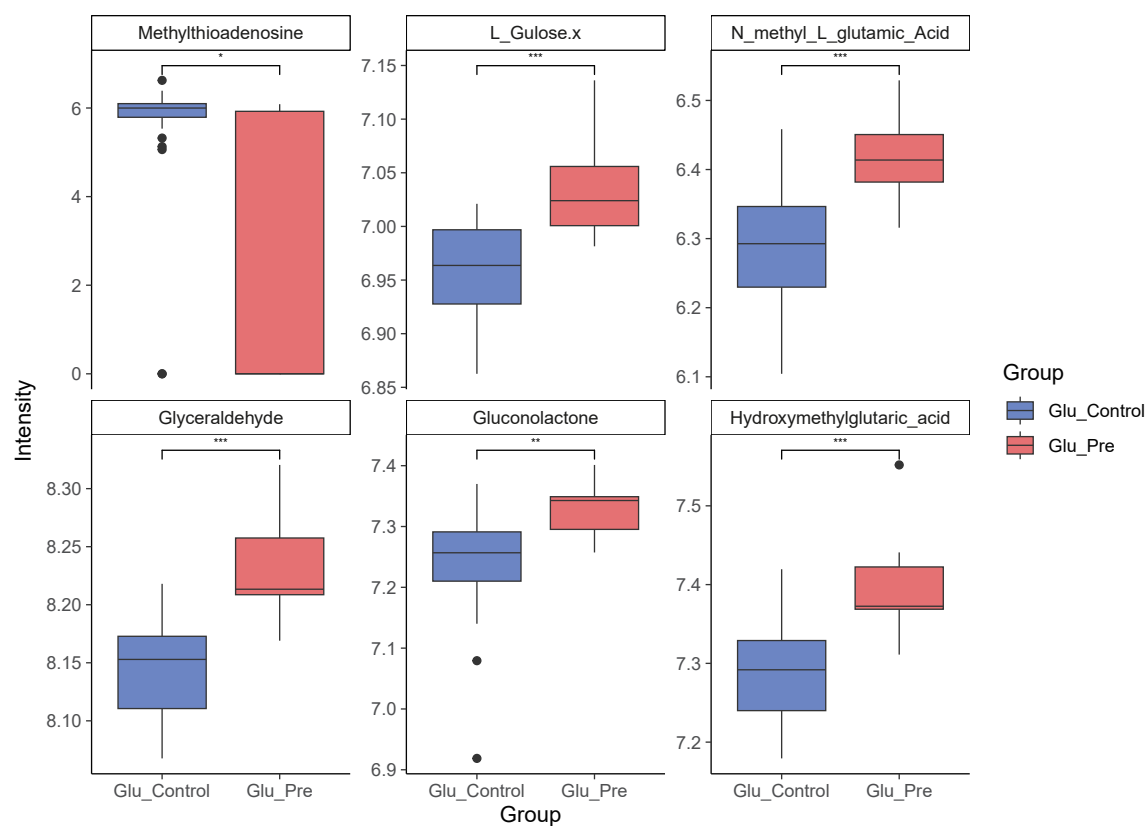

Supplement: Fig. S2 — Metabolic alterations in glucose pathway distinguish Pre-diabetes from Control groups without outlier sample. [file spectrum.01459-24-s0002.pdf]
