# Supplementary material for: Integrated multi-omics analysis reveals the functional signature of microbes and metabolomics in pre-diabetes individuals
Source: Microbiol Spectr. 2025 Jun 9;13(7):e01459-24. doi: 10.1128/spectrum.01459-24 (PMC12211063; doi:10.1128/spectrum.01459-24)
Supplement: Supplemental legends — Legends for Fig. S1 and S2. [file spectrum.01459-24-s0003.docx]

Figure S1 Metabolic Profiles Alterations Among the Diabetes group, Healthy control group, and Pre-diabetes group

(A,C) Partial least squares discriminant analysis (PLSDA) plot with samples’ identification, showing the discrimination between the diabetes and prediabetes groups (A), diabetes and normal groups (C) (red = prediabetes, blue = normal, green=diabetes).

(B, D) VIP scores derived from PLSDA loadings to show the importance of each metabolite and heatmap displays the abundance of metabolite in normal and prediabetes groups, with the rows that are scaled and clustered using Euclidean distance measurement. The color of the space ranges from red to blue. Redder spaces indicate a greater abundance of metabolite in the sample.

Figure S2 Metabolic Alterations in Glucose Pathway Distinguish Pre-diabetes from Normal groups without outlier sample

(A) Partial least squares discriminant analysis (PLSDA) plot with samples’ identification, showing the discrimination between the prediabetes groups and normal without outlier sample (red = prediabetes, blue = normal).

(B) VIP scores derived from PLSDA loadings to show the importance of each metabolite and heatmap displays the abundance of metabolite in normal and prediabetes groups, with the rows that are scaled and clustered using Euclidean distance measurement. The color of the space ranges from red to blue. Redder spaces indicate a greater abundance of metabolite in the sample.

(C) Boxplot show abundance of metabolites that play an important role in discrimination of prediabetes and normal groups. P values refer to Wilcoxon tests for statistical significance.
